# Screening and characterization of natural extracts as dual-functional regulators for cardiomyocyte regeneration and cardiac repair

**DOI:** 10.3389/fcvm.2025.1701482

**Published:** 2025-11-13

**Authors:** Jianfeng Liu, Chunyan Kuang

**Affiliations:** Department of Cardiovascular Diseases, Guizhou Provincial People’s Hospital, Guiyang, China

**Keywords:** cardiac differentiation, natural extracts, myocardial infarction, ganoderma lucidum water, wnt signaling pathway

## Abstract

**Objective:**

Cardiovascular diseases (CVDs), the leading cause of global mortality, demand novel strategies to enhance cardiac regeneration. While ongoing research has begun to address the critical need for novel therapies aimed at regenerating cardiomyocytes in the context of cardiovascular disease, existing pharmacological interventions remain insufficient. This study was designed to systematically evaluate natural extracts for their potential to enhance cardiomyocyte differentiation and facilitate cardiac repair.

**Methods:**

We performed an extensive literature review and identified 22 natural extracts associated with cardiac regeneration and stem cell differentiation. Through the use of a Tnnt2H2B-mCherry reporter mouse embryonic stem cell (mESC) line, we determined that Ganoderma lucidum water extract (GLW) are potent inducers of cardiomyocyte differentiation both *in vivo* and *in vitro*.

**Results:**

GLW extracts significantly increased the proportion of beating cardiomyocytes, upregulated cardiac-specific markers (e.g., Tnnt2, Myh6), and enhanced the expression of key transcription factors (Isl1, Nkx2.5, Gata4, Mef2c) during cardiac progenitor cell (CPC) specification. Mechanistically, GLW modulated the Wnt signaling pathway, which is critical for CPCs specification. *in vivo* research experiments have shown that GLW can treat mice with myocardial infarction (MI) and improve cardiac function,as evidenced by increased ejection fraction and fractional shortening, along with enhanced proliferation of cardiomyocytes. Notably, GLW, particularly at higher doses, and promoted structural maturation of cardiomyocytes.

**Conclusions:**

Our findings underscore the potential of GLW as promising candidates for dual-functional therapies targeting myocardial regeneration and injury mitigation, providing novel insights into natural product-based interventions for CVD management.

## 1 Introduction

Cardiovascular diseases (CVDs) remain the primary cause of mortality worldwide, accounting for approximately 18 million deaths annually, which represents 31% of total global fatalities (1, 2). Among these, ischemic heart disease (IHD), which encompasses a group of closely related syndromes resulting from myocardial ischemia (MI), is a leading contributor to patient morbidity and mortality (3–5). Despised significant advancements in modern medicine, which have improved symptom management, mitigated adverse cardiac remodeling, and reduced mortality through pharmacological therapies and surgical interventions (6, 7), several unresolved challenges persist. Notably, there remains an inability to fully compensate for myocardial damage caused by ischemia due to the limited regenerative capacity of terminally differentiated cardiomyocytes. This limitation results in irreversible cardiomyocyte loss during cardiac injury and heart failure (8). Consequently, the current approach to addressing cardiomyocyte loss resulting from ischemic heart disease primarily involves transplantation (9). Considering the scarcity of available donor hearts, the necessity for alternative therapeutic approaches becomes increasingly apparent.

Over the past two decades, researchers have consistently explored novel alternative therapies to mitigate myocardial loss caused by myocardial defects. Among these approaches, stem cell therapies have shown promise in terms of therapeutic feasibility and efficacy in regenerating injured myocardium (10–12). This is primarily attributed to the unique properties of stem cells, which include self-renewal, clonal expansion, and the ability to differentiate into specialized cell types, such as cardiomyocytes, under appropriate conditions (13). Significant progress has been made in treating cardiovascular diseases using stem cells derived from various sources, including embryonic stem cells (ESCs), induced pluripotent stem cells (iPSCs), and adult or somatic stem cells (13–17). However, the application of stem cell therapy for enhancing myocardial function continues to be limited by low implantation rates and suboptimal survival efficiency.

Natural products have historically played a pivotal role in pharmacotherapy, particularly in the treatment of cancer and infectious diseases (18, 19). For instance, plant-derived biocomposites have demonstrated biofilm-inhibitory effects through molecular docking and *in vitro* validation (20). Additionally, green-synthesized silver nanoparticles derived from pumpkin peel have exhibited radiosensitizing properties in cancer therapy (21). The therapeutic benefits and underlying mechanisms of various natural extracts in promoting the activation, proliferation, and differentiation of stem cells for myocardial repair (22–25) indicate that natural cardioprotective agents can be synergistically combined with stem cell therapies to provide more effective treatment options for patients with cardiovascular diseases. For instance, icariin, a major bioactive compound derived from epimedium, enhances the differentiation of mESCs into cardiomyocytes (26). Icariin can also increase endothelial nitric oxide synthase levels and nitric oxide production by activating the PI3K/pAkt/p-eNOS pathway and ERK pathway, thereby preventing endothelial dysfunction and alleviating atherosclerosis (27, 28). However, these studies lack a powerful tool for tracking the cardiomyocytes produced by ESCs, as well as identifying the specific stage at which natural products exert their functions. Moreover, the absence of *in vivo* verification reduces the credibility of the results.

In this study, the Tnnt2H2B-mCherry mESC reporter cell line was employed to evaluate the effects of various natural extracts on the mESCs derived cardiomyocyte. Specifically, natural extracts of GLW were found to promote the differentiation of mESCs into cardiomyocytes and enhance the expression CPC-specific transcription factors such as Isl1 (29), Nkx2.5 (30), and Mef2c (31). Gene Ontology analysis demonstrated that GLW extracts could regulate the Wnt signaling pathway and cardiac muscle development. *in vivo* experiments further confirmed that the extract of GLW was capable of mitigating myocardial infarction-induced damage, improving cardiac function, and promoting cardiomyocyte regeneration. Collectively, these results indicate that the screening and characterization of GLW provide promising alternatives for future clinical interventions in cardiovascular diseases.

## 2 Materials and methods

### 2.1 mESC culture

Mouse embryonic stem cell line R1 was obtained from ATCC (Cat# SCRC-1011). Prior to use, tissue culture dishes were coated with a 0.1% gelatin solution, and the cells were maintained in an undifferentiated state. The culture medium consisted of Dulbecco's Modified Eagle Medium (DMEM, Gibco) supplemented with 15% fetal bovine serum (FBS, Cat# 10091148, Gibco), 2 mM L-glutamine (Cat#25030,Gibco), 1%non-essential amino acids (Cat#11140-050,Invitrogen), 0.1 mM β-mercaptoethanol (Cat#21985,Gibco),100 μg/mL penicillin/streptomycin (Cat#1514012 2,Gibco), and 1,000 U/mL Leukemia inhibitory factor (Lif,Cat#50756-MNAH,sinobiological).

### 2.2 Preparation and pharmaceutical application of natural extracts

Extracts were selected based on their documented cardioprotective bioactivity and relevance to developmental pathways. All extracts conformed to standardized pharmacopeial criteria (e.g., HPLC quantification of marker compounds with ≥98% purity as shown in Supplementary Table S1). The experimental concentrations were calculated based on the dry weight (g/L) of the extracts, and aqueous or alcoholic solutions were prepared accordingly. A fresh stock solution of natural extracts at 40 g/L was prepared and dissolved in culture medium. The filtered and sterilized extracts were stored at −80°C and subsequently used according to the recommended drug concentrations outlined in Supplementary Table S1, with dilution in during cell culture.

### 2.3 MTT assay

Cellular MTT assays were conducted in a 96-well microplate (1 × 10^4^ cells/well in 100 μL of complete DMEM). Cells were incubated with various concentrations of extracts for 48 h. Subsequently, the medium was replaced with fresh medium containing a final concentration of 0.5 mg/mL of 3-(4,5-dimethylthiazol-2-yl) -2,5-diphenyltetrazolium bromide (MTT, Cat#ST1537, Beyotime), and the cells were further incubated for 4 h. Afterward, the medium was removed, and DMSO was added to dissolve the formazan crystals. Each group included six replicates. The optical density (OD) was measured using BioTek's Gen5™ Microplate Reader (BioTek, Winooski, VT, USA).

### 2.4 Reporter cell line establishment

Single-guide RNA (sgRNA) targeting the second exon of the Tnnt2 locus was designed using the CRISPR design tool (crispr.mit.edu) to induce homologous recombination and subsequently cloned into the pX330 plasmid. The sgRNA primers are listed in Supplementary Table S2. Additionally, the targeting donor vector was constructed with a 5′ homology arm-H2B-mCherry-polyA-Neo-3′ homology arm configuration (Supplementary Figure S1A). The target donor vector and the sgRNA/Cas9 expression plasmid pX330 were co-transfected into mESCs using the lipo8000 Transfection Reagent (Cat# C0533, Beyotime). The transfection mixture consisted of 125 µL of Opti-MEM containing 2.5 µg each of the sgRNA expression vector and the recombinant donor vector. After 48 h post-transfection, the cells were harvested and passaged. Stable clones expressing the neomycin were selected, and colonies resistant to G418 (500 μg/mL) were screened after two weeks of selection.

### 2.5 Immunofluorescence analysis

Cells were fixed with a 4% paraformaldehyde solution (Cat#P0099, Beyotime) for 20 min at room temperature and subsequently permeabilized in PBS containing 0.2% Triton X-100 for 20 min. After three washes with PBS, the cells were blocked with PBS supplemented with 10% fetal bovine serum (Cat# 10091148, Gibco) for 30 min at room temperature. The cells were then incubated overnight at 4°C with primary antibodies, including anti-Pou5f1 (1:500, Cat#sc-5279, Santa Cruz Biotechnology), anti-Sox2 (1:200, Cat#sc-365823, Santa Cruz Biotechnology), anti-SSEA-1 (1:100, Cat#sc-21702, Santa Cruz Biotechnology), and anti-mouse cardiac troponin T (1:500, Cat#sc-20025, Santa Cruz Biotechnology). Following this, the cells were incubated with FITC-conjugated goat anti-mouse IgG secondary antibodies (1:500,488 Alexa Fluor, Cat#ZF-0512, ZSGB-BIO or 594 Alexa Fluor, Cat#ZF-0513, ZSGB-BIO) for 1 h at 37°C. After immunofluorescence staining, the cells were respectively placed into a laser confocal microscope for photography and the expression of the staining was analyzed (including whether the staining was clear, whether the staining was complete, and whether each gene was fully expressed). Quantitative Analysis of Immunofluorescence Intensity and Colocalization Statistics Using ImageJ 1.54p.

### 2.6 mESCs differentiation

For the differentiation of mESCs, the suspension drop method was utilized to form embryoid bodies (EBs) as described in prior studies (32, 33). On day 0, approximately 800 mESCs were suspended in 20 μL of medium and cultured as hanging drops. After 3 days of differentiation, the resulting EBs were transferred to ultra-low attachment Petri dishes and cultured in suspension for an additional 2 days. On day 5, the EBs were plated onto gelatin-coated 24-well culture plates to observe beating EBs. The differentiation medium consisted of DMEM, supplemented with 20% fetal bovine serum (FBS), 0.1 mmol/L β-mercaptoethanol, and 1% nonessential amino acids, without Lif. The EB is classified as a true beating EB when the beat is detected in multiple locations (no fewer than two). During this period, the medium was refreshed every 2 days. Starting from day 6, the cells were treated with the recommended concentration of natural extracts. Counting Beating Frequency and Statistical Analysis Using Fluorescence Microscopy.

To modulate the Wnt signaling pathway during differentiation, the culture medium was supplemented with 20 ng/mL Wnt3a (CTRL group) for activation or 100 ng/mL DKK1 (GLW group) for inhibition on day 6. The experiment was terminated, and samples were harvested for analysis on induction day 15.

### 2.7 Transcriptome analysis and real-time PCR

RNA was extracted using TRIzol reagent (Cat#NG303M, Servicebio) and subsequently treated with DNase I (Cat#EN0521, Thermo Scientific) to remove any potential genomic DNA contamination. Complementary DNA (cDNA) was synthesized from 1 μg of total RNA using the cDNA Synthesis Kit (Cat#EG15133S, iScience). Real-time quantitative PCR was carried out using SYBR Premix ExTaqTM II (Cat#EG20117S/M, iScience) in accordance with the manufacturer's protocol. The expression levels of all target genes were normalized to beta-actin as an internal control gene. All experiments were performed in triplicate to ensure data reproducibility. Gene-specific primers were designed and validated based on the ΔΔCt method, as listed in Supplementary Table S2.

### 2.8 RNA-Seq analysis

For RNA-seq data analysis, clean reads were aligned to the mm10 mouse genome using HISAT2. Expression matrices and RPKM values were calculated using FeatureCounts. Differential gene expression across samples was analyzed using DESeq2, and heatmaps were generated using the R package pheatmap. Gene Ontology (GO) enrichment analysis was conducted using the R package clusterProfiler.

### 2.9 Alkaline phosphatase stain

The preparation of the alkaline phosphatase (AP) staining working solution was as follows: naphthol AS-MX phosphate (200 μg/mL, Cat#BCBT0082, Sigma-Aldrich), solid red TR salt (1 mg/mL, Cat# MKCD0656, Sigma-Aldrich), and Tris buffer (pH 8.2–8.4). Following cell fixation, cells were washed 3–5 times with a washing solution for 3–5 min each. Subsequently, an appropriate volume of AP staining working solution was added to the cells, followed by incubation at room temperature in the dark for 5–30 min until the desired color development was achieved. The AP staining solution was then removed, and the reaction was terminated by washing the cells 1–2 times with distilled water.

### 2.10 Grouping and administration of animal models of myocardial infarction in mice

A total of 60 male C57BL/6J mice (aged 6–8 weeks) were randomly assigned to four groups: sham-operated group (sham), myocardial infarction group (MI), MI with low-dose Ganoderma lucidum water extract group (MI + low GLW), MI with high-dose Ganoderma lucidum water extract group (MI + high GLW). Prior to MI induction, mice in the MI + low GLW, MI + h igh GLW groups were pre-treated with the respective natural82182 extracts for two weeks. The drug preparation concentration was as follows: GLW extract was administered at 600 mg/kg (high dose) or 300 mg/kg (low dose). The MI model was treated by permanently ligation the left anterior descending coronary artery (LAD), while mice in the Sham group underwent thoracotomy without ligation. Fourteen days post-surgery, transthoracic echocardiography was performed to assess cardiac function across all groups. Cine images of the left ventricle in both short- and long-axis views were acquired using the Vevo 2100 high-resolution ultrasound system. Mice were maintained under light isoflurane anesthesia, and electrocardiographic (ECG) signals were continuously monitored via limb electrodes. M-mode echocardiography was used to measure end-diastolic volume (182EDV) and end-systolic volume (ESV), from which left ventricular ejection fraction (EF) and other cardiac parameters were derived using the following formulas: EF% = (EDV − ESV)/EDV × 100, SV = EDV − ESV, and CO = SV × beats per minute (dpm). The approval date was November 2024. At the endpoint of the study, euthanasia was performed under deep anesthesia induced by inhalation of 5% isoflurane, followed by an intraperitoneal injection of an overdose of sodium pentobarbital (200 mg/kg). This method is consistent with the recommendations of the American Veterinary Medical Association (AVMA) Guidelines for the Euthanasia of Animals (2020). All efforts were made to minimize suffering.

### 2.11 Sirius red staining

To assess cardiac fibrosis, heart sections from the MI and GLW groups of myocardial infarction model mice were subjected to Sirius red staining. Briefly, after deparaffinization and rehydration, the sections were incubated with Sirius red stain for 1 h. After a brief wash in running water, the sections were dehydrated, cleared, and cover-slipped with neutral balsam. Subsequently, the collagen area (fibrosis index) was measured from the stained sections using ImageJ software. Finally, the average collagen area per group (*n* = 3) was determined, and intergroup differences were statistically compared.

### 2.12 Cardiac tissues immunofluorescence staining

Cardiac tissues were fixed in 4% paraformaldehyde for 24–48 h and subsequently processed for paraffin embedding. Serial sections of 4 μm thickness were obtained using a microtome and mounted onto glass slides. For dewaxing, antigen retrieval, and blocking, paraffin-embedded sections were subjected to sequential treatment with xylene and graded ethanol solutions. Antigen retrieval was performed by heating the sections in citrate buffer (pH 6.0) within a pressure cooker for 3 min, followed by cooling at room temperature for 20 min. Sections were then permeabilized with 0.1% Triton X-100 for 10 min and blocked with an immunostaining blocking buffer containing 5% normal goat serum and 0.1% Triton X-100 in PBS for 1 h at 37°C. Primary antibodies were prepared at optimal concentrations in the blocking buffer as follows: mouse anti-cardiac troponin T (1:200, Cat#15513-1-AP, Proteintech), rabbit anti-cardiac troponin T (1:200, Cat# AF0078, Beyotime), rabbit anti-Ki67 (1:100, Cat# ab15580, abcam), and rabbit anti-phospho-histone H3 (Ser10) (1:200, Cat# AF1180,Beyotime),anti-smooth muscle antibodies(1:100,TX20243, abcam). The tissue sections were incubated with these primary antibodies overnight in a humidified chamber at 4°C. Following incubation, sections were washed three times with PBS and incubated with appropriate secondary antibodies for 1–2 h at room temperature in the dark. The secondary antibodies used were Alexa Fluor 488-labeled goat anti-rabbit IgG (H + L), Alexa Fluor 488-labeled goat anti-mouse IgG (H + L), Alexa Fluor 647-labeled goat anti-rabbit IgG (H + L), and Alexa Fluor 647-labeled goat anti-mouse IgG (H + L) (all from Beyotime, diluted 1:200 in blocking buffer). Nuclear Counterstaining and Imaging: Nuclear counterstaining was conducted using DAPI (10 μg/mL) for 10 min, followed by three rinses with PBS. Slides were subsequently mounted and examined under a laser confocal microscope (Olympus Fluoview FV3000, Tokyo, Japan). Immunofluorescence images were captured using the Olympus Fluoview FV3000 version 3C Acquisition Software. Quantification of Positive Cells: To quantify Ki67^+^ or pH3^+^ cardiomyocytes (CMs), heart sections from each animal were analyzed. At least five distinct fields per section were evaluated at 200–400× magnification. The average number of positive cells per field was calculated and presented as the mean ± SEM. All quantifications were performed in a blinded manner to ensure unbiased results. Quantitative Analysis of Immunofluorescence Intensity and Colocalization Statistics Using ImageJ 1.54p.

### 2.13 Statistical analysis

Statistical Analysis of Sirius Red Staining, Immunofluorescence Intensity, and Colocalization Using ImageJ 1.54p. GraphPad Prism 10 (version 10.4.0) software was used for data analysis. Dates are shown as mean ± SEM. Unpaired Student's *t*-test was used to compare differences between the two groups. Comparisons between multiple groups were analyzed using one-way ANOVA with Tukey's analysis. *P < 0.5, **P < 0.01 and ***P < 0.001 were considered statistically significant.

## 3 Results

### 3.1 Construction and validation of the Tnnt2-H2b-mCherry + reporter cell line

**Figure 1:**
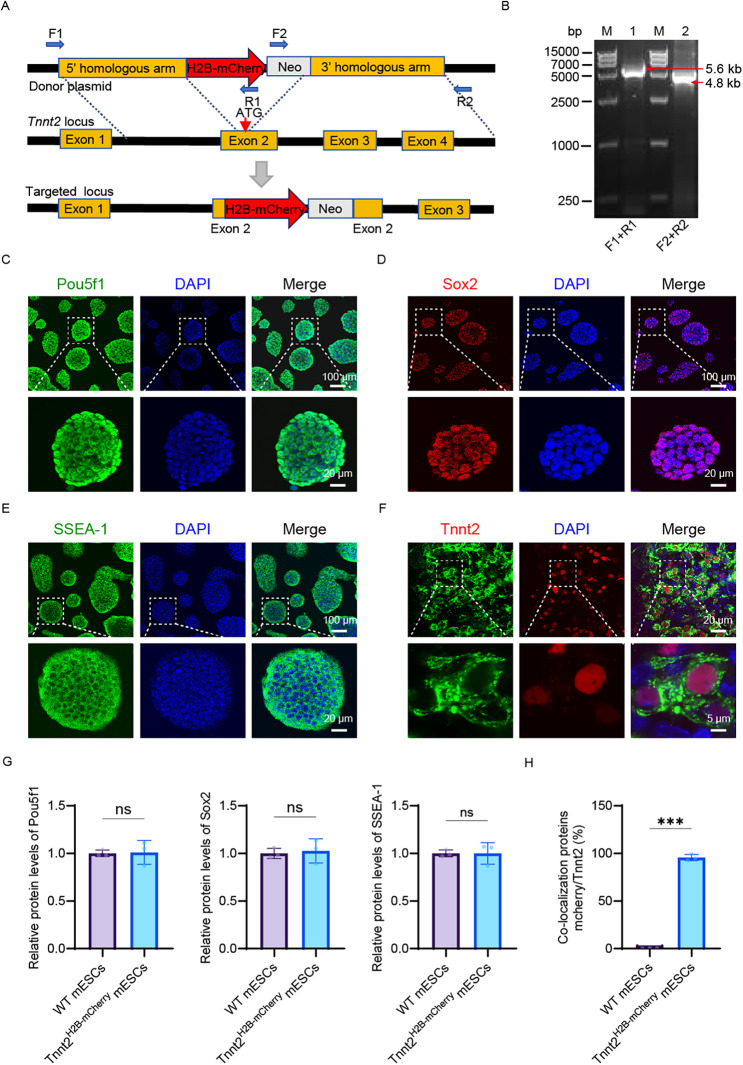
Construction and characterization of the Tnnt2-H2B-mCherry reporter mESC line. **(A)** Schematic representation of the construction process for the Tnnt2H2B-mCherry reporter cell line. **(B)** PCR validation of the 5′ and 3′ homology arms in the reporter cell line. Lane 1: 5′ homology arm; Lane 2: 3′ homology arm. Lane M: DNA ladder. **(C–E)** Pluripotency assessment of Tnnt2-H2B-mCherry mESCs. Immunofluorescence staining for the core pluripotency markers Pou5f1 **(C)**, Sox2 **(D)**, and the surface marker SSEA-1 **(E)** Nuclei are counterstained with DAPI (blue). The merged images confirm the expression of these markers in the reporter cell line. Scale bar in main panels = 100 *μ*m; scale bar in magnified insets = 20 *μ*m. **(F)** Cardiomyocyte differentiation analysis. Immunostaining for cardiac Troponin T (Tnnt2, green) in Tnnt2-H2B-mCherry-derived cardiomyocytes at day 15 of differentiation. Nuclei are stained with DAPI (blue). The merged image shows the localization of Tnnt2. Scale bar in main panels = 20 *μ*m; scale bar in magnified insets = 5 *μ*m. **(G)** Quantitative analysis of pluripotency marker expression. Relative protein expression levels of Pou5f1, Sox2, and SSEA-1 in wild-type (WT) mESCs and Tnnt2-H2B-mCherry mESCs, as determined by immunofluorescence intensity quantification. Statistical analysis shows no significant difference (ns), indicating that the genetic modification did not alter the pluripotent state of the cells. **(H)** Quantification of reporter specificity in cardiomyocytes. Analysis of the co-localization between the mCherry reporter signal and endogenous Tnnt2 immunostaining in differentiated cardiomyocytes. The graph demonstrates a high degree of co-localization, validating the specificity of the Tnnt2-driven mCherry reporter. *G–H Data are presented as mean* *±* *SEM. Statistical analysis was performed using unpaired Student’ s t-test. n* *=* *3 per group; (ns, not significant, p* *>* 0.05, *p *<* 0.05, **p *<* 0.01, ***p *<* *0.001).*

We generated a reporter cell model using the CRISPR/Cas9 system to knock-in a reporter gene at the Tnnt2 locus, a strategy outlined in Figure 1A. The Tnnt2 gene (troponin T type 2), which enables cardiomyocyte-specific expression, was fused with an H2B-mCherry nuclear localization signal to facilitate the visualization of cardiomyocytes. Targeted mouse embryonic stem cells (mESCs) were identified via long-fragment PCR using primers (F1–R1 and F2–R2) located outside the 5′ and 3′ homology arms, respectively (Figure 1B, Supplementary Figure S1B). To avoid the absence of Tnnt2 protein that would result from a homozygous knock-in, we selectively isolated heterozygous knock-in cells (Tnnt2H2B-mCherry/+) using PCR (Supplementary Figure S1C). Clone #10, confirmed as heterozygous, was selected for further genomic analysis. Sanger sequencing verified the precise integration of the reporter at the target site (Supplementary Figure S1D), and this cell line was designated as Tnnt2H2B-mCherry for subsequent studies.

The resulting knock-in cell line exhibited typical clonal morphology (Figures 1C–E, Supplementary Figure S1E) and maintained normal expression of key pluripotency markers, including nuclear Pou5f1 (Oct3/4), Sox2, and SSEA-1 (Figures 1C–E). The reporter cells also showed active alkaline phosphatase (AP) expression, a marker for undifferentiated mESCs (Supplementary Figure S1E). Quantitative PCR (qPCR) analysis revealed no statistically significant differences in the expression levels of pluripotency genes (Pou5f1, Sox2, Nanog) between the reporter and control cell lines (Supplementary Figure S1F). Consistent with this, immunofluorescence analysis confirmed that the Tnnt2^H2B−mCherry^ cells retained pluripotency comparable to the parental mESCs (WT) at the protein level (Figure 1G), indicating that the reporter integration did not disrupt the expression of pluripotency-associated genes. This precise integration approach aligns with modern methods for engineering reporter cell lines that minimize genomic disruption.

To validate the reporter's ability to recapitulate endogenous Tnnt2 activity, we differentiated the Tnnt2^H2B−mCherry^ cells into cardiomyocytes. Immunostaining confirmed that the H2B-mCherry fusion protein was localized to the nucleus and co-expressed with endogenous Tnnt2 (Figure 1F). Quantitative analysis demonstrated nearly complete co-localization of mCherry and Tnnt2 in the reporter cell line, in contrast to the WT which showed no mCherry signal (Figure 1H). Furthermore, the differentiated Tnnt2^H2B−mCherry^ cardiomyocytes exhibited spontaneous contractions, a characteristic functional property (Movie 1). In summary, the successful generation of this reporter cell line provides a robust tool for elucidating the spatiotemporal expression pattern of endogenous Tnnt2 and enables real-time tracking and purification of cardiomyocytes. The use of quantitative single-cell reporters, as demonstrated here, is a powerful approach for characterizing gene expression with high sensitivity and resolution in developmental systems.

### 3.2 GLW promotes cardiomyocyte induction

**Figure 2:**
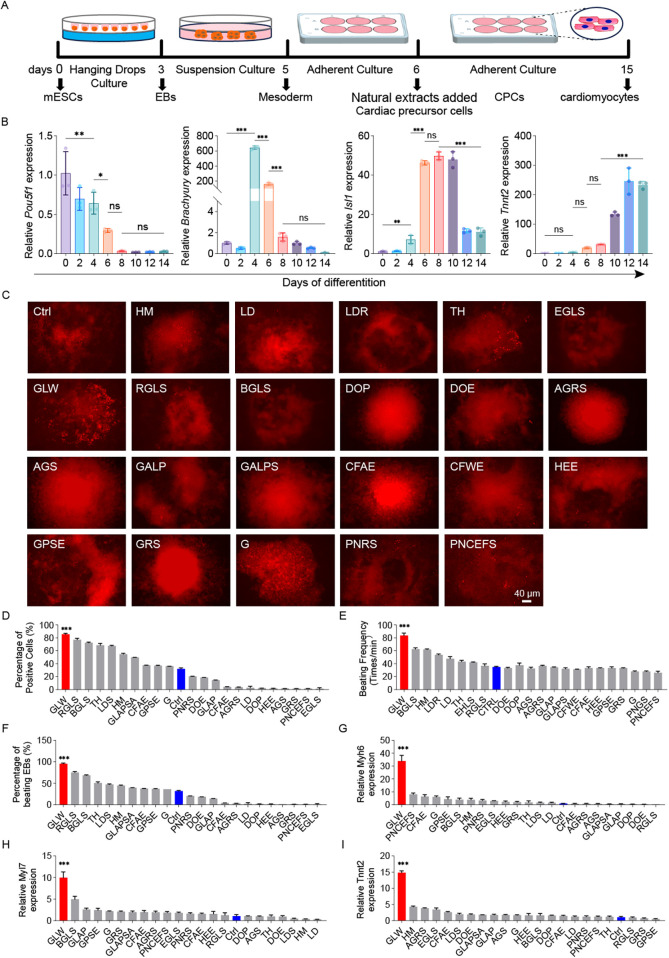
GLW enhances cardiomyocyte differentiation from mESCs. **(A)** Schematic of the EB-based suspension culture protocol used for cardiomyocyte differentiation. **(B)** Time-dependent expression of marker genes (*Pou5f1*, *Brachyury/T*, *Isl1*, *Tnnt2*) during differentiation, as measured by qPCR. **(C)** Representative fluorescence images of mCherry-positive cardiomyocytes induced by 23 candidate extracts and the control (ctrl) on day 10. Scale bar = 40 *μ*m. *n* = 3 per group. **(D)** Quantitative analysis of mCherry-positive cell proportions among the 23 candidate extracts and ctrl. **(E)** Beating rates of cardiomyocytes (beats per minute) induced by the 23 extracts and ctrl. **(F)** Proportion of contracting EBs recorded on day 15 across the 23 extracts and ctrl. **(G–I)** RT-qPCR analysis of cardiac-specific gene expression (*Myh6*, *Tnnt2*, *Myl7*) for the 23 extracts and ctrl. *B, D-I Data are presented as mean* *±* *SEM. statistical significance was determined using one-way ANOVA followed by Tukey's post hoc test. n* *=* *3 per group; (ns, not significant, p* *>* 0.05, *p *<* 0.05, **p *<* 0.01, ***p *<* *0.001).*

We employed a classical embryoid body (EB)-based suspension culture system to induce cardiomyocyte differentiation from mouse embryonic stem cells (mESCs) (Figure 2A). The differentiation process was monitored by analyzing the expression of key marker genes, which defined four distinct developmental stages: undifferentiated mESCs (*Pou5f1/Oct3/4*), mesodermal cells (*Brachyury*), cardiac progenitors (*Isl1*), and functional cardiomyocytes (*Tnnt2*). Quantitative PCR (qPCR) confirmed the sequential activation of these markers over time (Figure 2B). To evaluate the impact of natural extracts on cardiomyocyte induction, we first assessed their potential cytotoxicity. An MTT assay was used to determine the relative viability of Tnnt2^H2B−mCherry^ reporter cells after 48 h of treatment with various extract concentrations (Supplementary Figure S2). Based on these results (Supplementary Figures S2, S3 and Table S1), an optimal concentration that maintained 100% cell viability was selected for subsequent differentiation experiments.

After adding various extracts, we evaluated their effects on cardiac differentiation (Figure 2C). Quantitative analysis of the percentage of fluorescent cells post-induction and the proportion of mCherry-positive cells (Figure 2D) revealed that GLW treatment generated a significantly higher number of mCherry-positive cells. Furthermore, approximately 90% of EBs in the GLW-treated group exhibited spontaneous contractions (Figure 2E), and the beating rate of cardiomyocytes reached around 90 beats per minute (Figure 2F), both of which were markedly higher than those in the control group and other extract-treated groups. These findings indicate a robust cardiomyocyte induction capacity of GLW. To further validate the promotive effect of GLW on cardiomyocyte differentiation, we examined the expression levels of cardiac-specific genes, including *Tnnt2*, *Myh6*, and *Myl7* (Figures 2G–I). Consistent with the functional induction results observed in beating EBs, the aqueous extract of GLW significantly upregulated the mRNA expression of *Tnnt2*, *Myh6*, and *Myl7*. Collectively, these results suggest that GLW possesses potential promoting effects on the induction of cardiomyocytes derived from mESCs.

### 3.3 GLW specifically promotes the CPC formation during the cardiomyocyte induction

**Figure 3:**
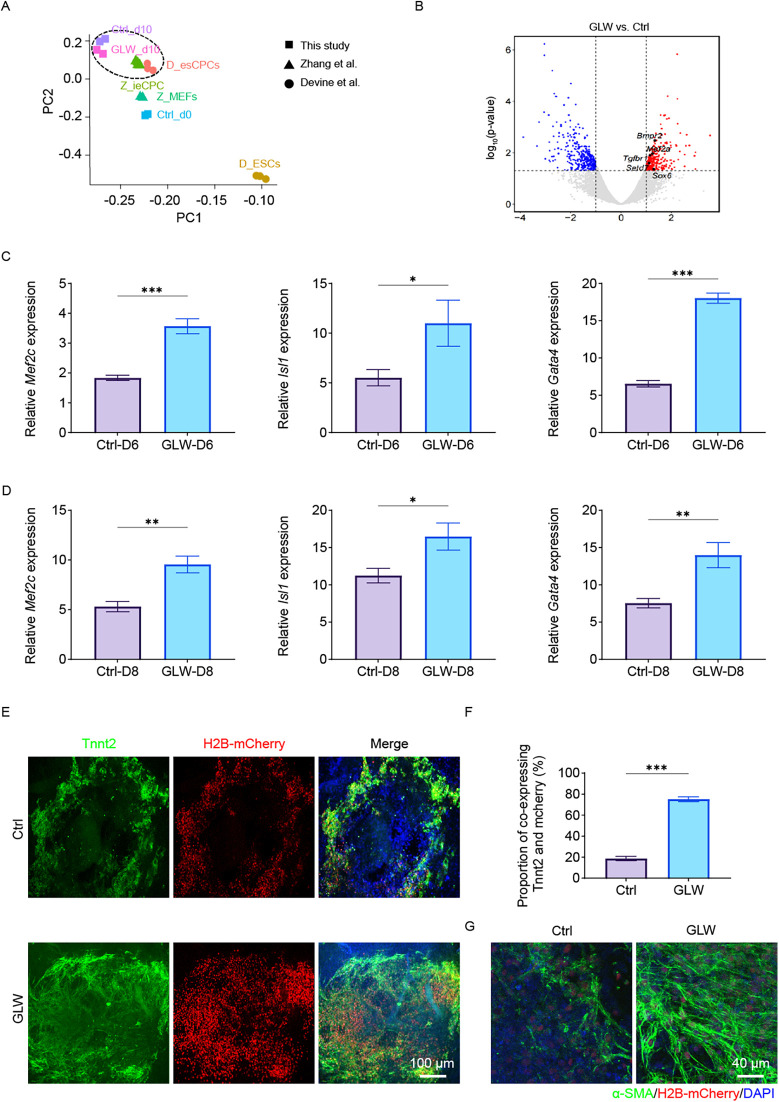
GLW promotes cardiac precursor cell induction and cardiomyocyte maturation. **(A)** Principal component analysis (PCA) of RNA-Seq data from control (Ctrl) and GLW-treated samples at day 10 of differentiation, projected alongside reference cell types (Z-MEF, D-ESC, Z-iCPC, Z-CPC). The GLW-d10 samples cluster within the CPC stage. **(B)** Volcano plot showing differentially expressed genes (DEGs) between GLW and Ctrl groups at day 10. Key upregulated genes related to heart development (Tgfbr2, Bmpr2, Mef2a, Setd7, Sox6) are highlighted. (C and D)RT-qPCR analysis confirming the upregulation of CPC markers (Isl1, Mef2c) and the early cardiac marker Gata4 in GLW-treated cells at day 6 **(C)** and day 8 **(D)**. **(E)** Representative immunofluorescence images of day 15 cardiomyocytes stained for cardiac troponin T (Tnnt2, green) and nuclei (DAPI, blue). H2B-mCherry labels differentiated cardiomyocytes. Yellow areas indicate Tnnt2/mCherry double-positive cells. Scale bar, 100 *μ*m. **(F)** Quantitative analysis of the proportion of Tnnt2 and mCherry co-expressing cells. Data show a significant increase in the GLW group compared to the Ctrl group. **(G)** Immunofluorescence staining for alpha-smooth muscle actin (α-SMA, green) in differentiated cardiomyocytes, showing clearer and more structured filaments in the GLW group. Nuclei are stained with DAPI (blue). Scale bar, 40 μm. *C, D, F Data are presented as mean* *±* *SEM. Statistical significance was determined by Student's t-test n* *=* 3 per group; (ns, not significant, *p *<* 0.05,** p *<* 0.01, ***p *<* *0.001).*

To further elucidate the roles of GLW in mESC-induced cardiomyogenesis, RNA-seq analysis was performed on samples differentiated up to day 10 and compared with established cardiac progenitor cell (CPC) datasets (34–36). Principal Component Analysis (PCA) demonstrated that the day 10 control, GLW-induced cells clustered within the CPC stage (Figure 3A). Differential gene expression analysis identified a suite of genes significantly upregulated by GLW, including key transcription factors and signaling components essential for heart development such as Tgfbr2, Bmpr2, and Mef2a (Figure 3B). We validated this enhanced CPC signature at the transcriptional level, confirming that GLW significantly boosted the expression of established CPC markers (Isl1, Mef2c) and the early cardiac determinant Gata4 at both day 6 and day 8 (Figures 3C,D). Together, these data demonstrate that GLW instructs a transcriptome conducive to cardiac lineage specification.

By day 15, immunostaining for Tnnt2 revealed a substantially greater yield of Tnnt2^+^/mCherry^+^ cardiomyocytes in GLW-treated cultures compared to controls (Figure 3E). quantification confirmed that the proportion of Tnnt2+/mCherry+ double-positive cardiomyocytes was significantly higher in the GLW-treated group (∼75%) compared to the control group (∼20%) (Figure 3F). Furthermore, GLW treatment promoted the coordinated differentiation of smooth muscle cells, indicated by enhanced and more structured α-smooth muscle actin (α-SMA) filaments (Figure 3G). Thus, GLW not only efficiently commits mESCs to the cardiac lineage but also fosters the development of structurally mature and integrated cardiac tissue.

### 3.4 GLW exhibits a more pronounced effect on cardiomyocyte induction

**Figure 4:**
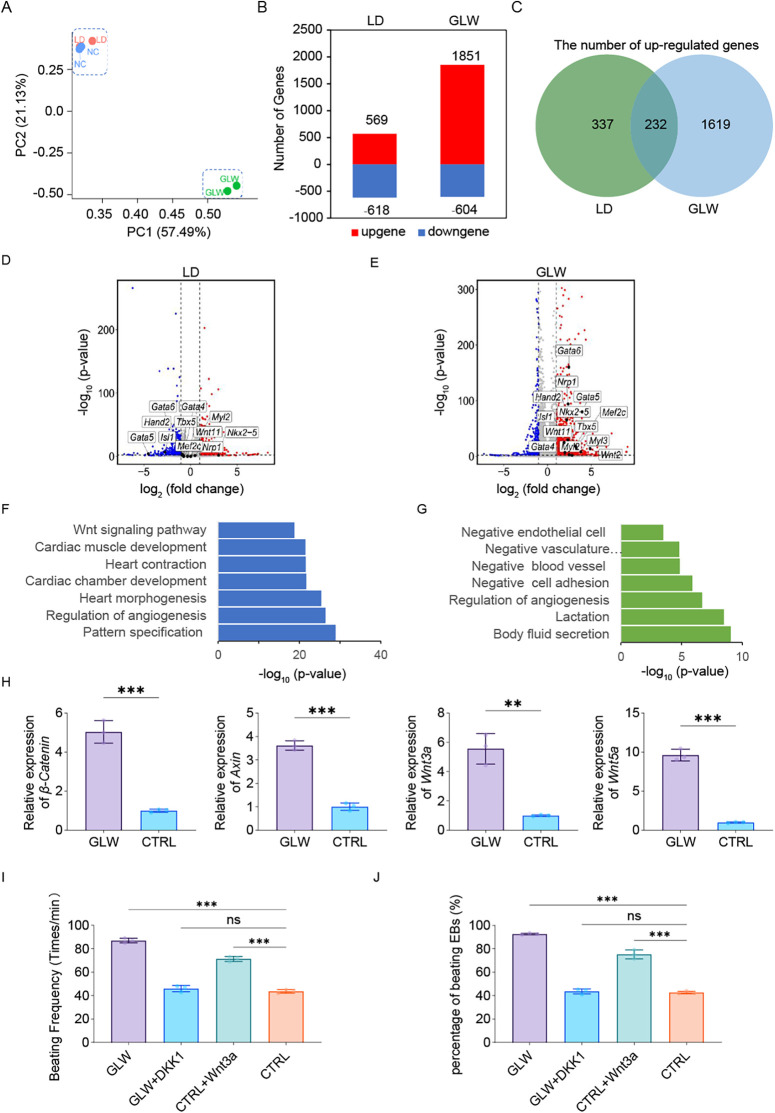
GLW promotes cardiomyocyte differentiation of ESCs by activating the Wnt signaling pathway. **(A)** Principal component analysis (PCA) of RNA-Seq data from day 15 samples under control, LD, and GLW treatment conditions (*n* = 2 biologically independent samples per group). **(B)** Bar plot showing the number of upregulated and downregulated genes in LD- and GLW-treated groups compared to control. **(C)** Venn diagram illustrating the overlap of upregulated genes between LD and GLW treatments. **(D,E)** Volcano plots displaying differentially expressed genes in the **(D)** LD and **(E)** GLW groups with data collected at day 15. Key cardiac transcription factor *Gata4* is highlighted. **(F,G)** Gene Ontology (GO) enrichment analysis of upregulated genes in the **(F)** GLW and **(G)** LD groups. Selected significantly enriched terms are shown. **(H)** Quantitative RT-PCR analysis of the expression of key Wnt signaling pathway genes (*CTNNB1, Axin1, Wnt3a, Wnt5a*) in the control and GLW groups. *Data are presented as mean* *±* *SEM. Statistical significance was determined by Student's t-test n* *=* 3 per group; (** p *<* 0.01, ***p *<* *0.001).* **(I,J)** Functional validation of Wnt pathway involvement. **(I)** quantitative analysis of beating frequency and **(J)** the proportion of beating cells following treatment with the Wnt agonist Wnt3a (20 ng/mL, from day 6 to day 15) in the control group, or the Wnt inhibitor Dkk1 (50 ng/mL, from day 6 to day 15) in the GLW group. *Data are presented as mean* *±* *SEM. statistical significance was determined using one-way ANOVA followed by Tukey's post hoc test. n* *=* *3 per group; (ns, not significant, p* *>* 0.05, ***p *<* *0.001).*

To gain a comprehensive understanding of the promoting effects of GLW on ESC-derived cardiomyocyte differentiation and its underlying mechanisms during induction, we performed RNA-seq analysis on day 15 samples, with the LD group serving as an inhibitory control. Principal component analysis (PCA) revealed that the control and LD groups exhibited greater similarity, while the two GLW groups showed closer clustering, indicating distinct transcriptomic profiles induced by GLW treatment. Each group included three biological replicates (Figure 4A).

Differential gene expression analysis identified 569 upregulated and 618 downregulated genes in the LD-treated group, while the GLW-treated group showed 1851 upregulated and 604 downregulated genes (Figure 4B). Among the upregulated genes, 232 were common to both GLW and LD treatments (Figure 4C), including cardiac-related transcription factors such as Gata4, an early cardiac marker (Figures 4D,E).

GO enrichment analysis of upregulated genes indicated that GLW significantly enriched terms associated with ventricular development, cardiac contraction, and myocardial development (Figure 4F). In contrast, LD-induced genes were enriched in GO categories such as “negative regulation of vascular morphogenesis,” “negative regulation of vascular system development,” “negative regulation of endothelial cell migration,” and “endocytosis,” suggesting an inhibitory role of LD in cardiomyocyte differentiation (Figure 4G). Notably, GLW was implicated as a regulator of the Wnt signaling pathway (Figure 4F), which is well-established in cardiac development (37–39).

Validation of the Wnt pathway involvement showed that the expression of key genes—CTNNB1, Axin1, Wnt3a, and Wnt5a—was significantly elevated in the GLW group compared to controls (Figure 4H). Functional confirmation was obtained through agonist and inhibitor assays: supplementation with Wnt agonist Wnt3a in the control group increased both the beating frequency and ratio of cardiomyocytes, whereas adding the Wnt inhibitor Dkk1 to the GLW group reduced these parameters (Figures 4I,J). These results collectively indicate that GLW enhances cardiomyocyte differentiation by activating the Wnt signaling pathway.

### 3.5 GLW improve cardiac repair in mouse myocardial infarction model

**Figure 5:**
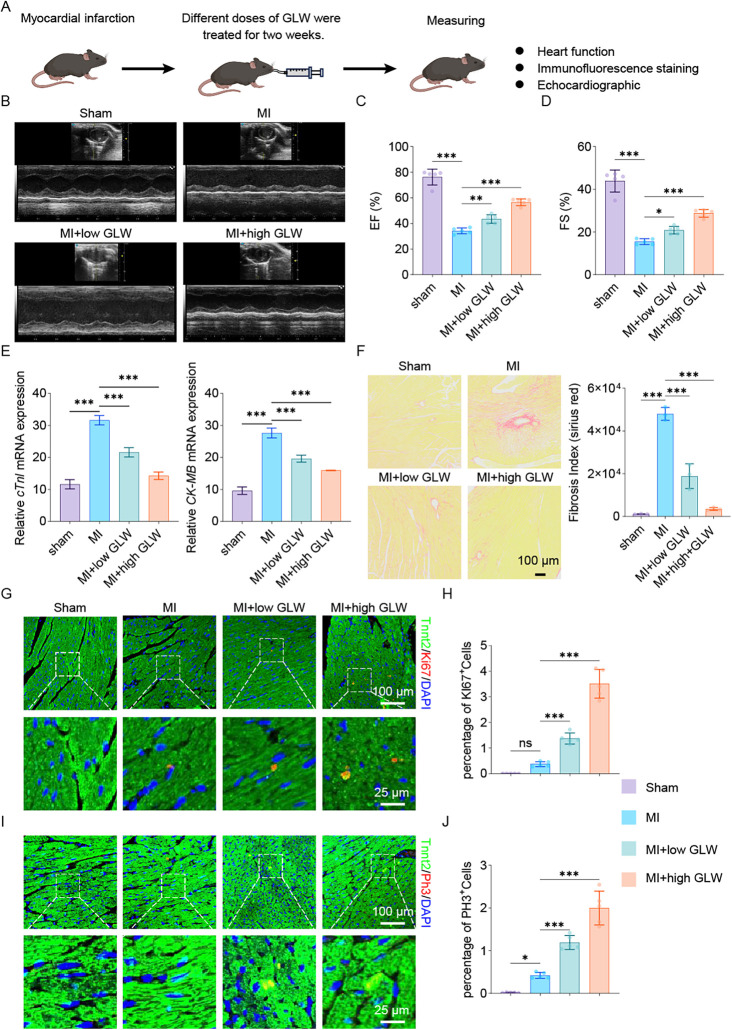
GLW treatment improves cardiac function and promotes repair after myocardial infarction in mice. **(A)** Schematic diagram of the experimental timeline. Mice were subjected to MI surgery or sham operation, followed by treatment with varying doses of GLW for two weeks. **(B–D)** Assessment of cardiac function by echocardiography. **(B)** Representative echocardiographic images. **(C,D)** Quantitative analysis of ejection fraction (EF%, **C**) and fractional shortening (FS%, **D**) showing significant impairment in the MI group compared to the Sham group, which was ameliorated by GLW treatment, particularly at the high dose. **(E)** mRNA expression levels of myocardial injury markers cardiac troponin I (cTnI) and creatine kinase MB isoenzyme (CK-MB). MI induced a significant upregulation of both markers, which was suppressed by GLW in a dose-dependent manner. Total RNA was extracted from heart samples collected from the myocardial infarction model after 2 weeks of intervention. **(F)** Representative images and quantitative analysis of Sirius Red staining, demonstrating collagen deposition. GLW treatment significantly inhibited MI-induced cardiac fibrosis. **(G)** Assessment of cardiomyocyte proliferation by Ki67 immunofluorescence. *Top panels*: Lower-magnification views of the infarct border zone. *Bottom panels*: Higher-magnification views of the areas indicated by the dashed boxes above, showing co-staining of the cardiomyocyte marker cardiac troponin T (Tnnt2, green) and the proliferation marker Ki67 (red). Nuclei are labeled with DAPI (blue). Co-localization of Tnnt2 and Ki67 signals (yellowish in merge) indicates proliferating cardiomyocytes. **(H)** Quantitative analysis of the percentage of Ki67-positive cardiomyocytes. **(I)** Assessment of cardiomyocyte proliferation by phosphorylated histone H3 (pH3) immunofluorescence. Layout and staining are identical to **(G)**, with Tnnt2 (green) and pH3 (red). **(J)** Quantitative analysis of the percentage of pH3-positive cardiomyocytes. *Data are presented as mean* *±* *SEM. statistical significance was determined using one-way ANOVA followed by Tukey's post hoc test. n* *=* *3 per group; (ns, not significant, p* *>* 0.05, *p *<* 0.05, **p *<* 0.01, ***p *<* *0.001).*

To investigate the effect of GLW on cardiac repair following myocardial infarction (MI), we established a mouse MI model and administered different doses of GLW for two weeks (Figure 5A). Cardiac function was first assessed by echocardiography. Compared to the Sham group, mice in the MI model group exhibited a significant impairment in left ventricular function, indicated by markedly reduced ejection fraction (EF%) and fractional shortening (FS%) (Figures 5B–D). Treatment with GLW, particularly at the high dose, effectively ameliorated these functional parameters.

Subsequently, we measured the mRNA expression levels of myocardial injury markers. The mRNA expression of cardiac troponin I (cTnI) and creatine kinase MB isoenzyme (CK-MB) was significantly upregulated in the myocardial tissue of the MI group compared to the Sham group (Figure 5E). GLW treatment resulted in a dose-dependent reduction in the expression of both markers, indicating that GLW attenuated MI-induced myocardial injury.

Given that MI often leads to pathological cardiac fibrosis, we evaluated collagen deposition using Sirius Red staining. The collagen fraction (fibrosis index) was significantly higher in the MI group than in the Sham group (Figure 5F). GLW treatment markedly inhibited collagen deposition, thereby reducing the extent of myocardial fibrosis.

To elucidate whether the protective effect of GLW is associated with the promotion of cardiomyocyte proliferation, we further examined the expression of the proliferation marker Ki67 and phosphorylated histone H3 (pH3). Immunofluorescence staining results (Figures 5G–J) revealed that, compared to the MI group, the number of cardiomyocytes (labeled by α-actinin) that were positive for Ki67 (Figures 5G,H) and pH3 (Figures 5I,J) was significantly increased in the GLW-treated groups. This suggests that GLW promotes cardiomyocyte proliferation.

In summary, these results demonstrate that GLW improves cardiac function, reduces myocardial injury and fibrosis, and promotes cardiomyocyte proliferation in mice with MI. The underlying mechanism may involve the enhancement of cardiomyocyte proliferation.

## 4 Discussion and conclusion

In this study, we sought to identify novel natural extracts that could promote the differentiation of stem cells into cardiomyocytes and enhance cardiac function following myocardial infarction. To achieve this objective, we employed the Tnnt2H2B-mCherry reporter cell line, which specifically tracks and visualizes cardiomyocyte differentiation during ESC-derived induction, for screening potential inducers.

Emerging studies highlight the significant therapeutic potential of natural compounds in disease management. A broad spectrum of natural compounds has garnered substantial attention in various experimental models and clinical trials related to cardiovascular diseases, including icariin, resveratrol, ginseng, lupinine, ursolic acid, and curcumin, as previously reviewed (40). To further identify additional suitable compounds for improving heart function, we systematically screened 22 natural extracts associated with cardiac regeneration and stem cell differentiation and using the Tnnt2H2B-mCherry reporter cell line developed in our laboratory.

In this study, we demonstrated that GLW extract significantly enhanced the expression of cardiac progenitor-associated transcription factors Isl1, Mef2c, and Gata4 during induction. Furthermore, GLW-induced genes were involved in DNA replication, chromosome organization, and cell-cycle regulation, thereby facilitating gene expression activation and promoting cell proliferation. This extract could potentially complement cell-based therapies by enhancing the viability and integration of transplanted cardiomyocytes, as evidenced by reduced fibrosis and increased proliferative markers. Moreover, natural extracts such as GLW are amenable to large-scale production and have a long history of safe use in traditional medicine, which may reduce regulatory hurdles for clinical translation. Finally, it was proved that the GLW extract promoted the differentiation of cardiomyocytes by activating the Wnt signaling pathway.

Some literature indicates that Ganoderma lucidum extract has therapeutic potential in improving endothelial dysfunction, regulating angiogenesis and its regeneration, and reducing atherosclerosis. Ganoderma lucidum extract significantly reduces serum total cholesterol (TC), triglycerides (TG), and low-density lipoprotein (LDL), thereby reducing lipid deposition and macrophage infiltration in atherosclerotic plaques. Mechanistically speaking, it inhibits the NF-*κ*B signaling pathway, thereby suppressing the secretion of pro-inflammatory cytokines (TNF-α, IL-6, IL-1β) and enhancing the anti-inflammatory cytokine IL-10, ultimately slowing down the development of cardiovascular diseases (41). Extracts related to Ganoderma lucidum exert anti-vascular aging and its regenerative effects by regulating the cell cycle and age-related secretory phenotype (SASP), reducing DNA damage, alleviating oxidative stress, improving mitochondrial function and regulating metabolic levels. In addition, it improves atherosclerosis and vascular calcification related to vascular aging in the body. Mechanistically speaking, Sirt 7 inhibitors can aggravate the senescence and calcification of vascular smooth muscle cells（VSMCs）, while the anti-aging effect of Ganoderma lucidum triterpenoids and their inhibitory effect on the senescence and calcification of VSMCs are both blocked. It was innovatively discovered that Sirt 7 interacts with Keap 1, promoting the deacetylation of Keap 1, thereby facilitating the dissociation of Keap 1-NRF 2, and further enhancing the nuclear translocation and activation of Nrf 2, thus generating new cells to protect the cardiovascular system (42).

In summary, our findings indicate that GLW serve as potent inducers of cardiomyocyte differentiation, and demonstrating a particularly significant enhancement in the differentiation of cardiomyocytes derived from mESCs and it promoted the differentiation of cardiomyocytes by activating the Wnt signaling pathway. The *in vivo* data further confirm that GLW extract exert protective effects against myocardial infarction and effectively alleviate post-MI myocardial injury. This study not only advances our understanding of the myocardial regeneration mechanism but also establishes an experimental basis for the development of a cardiovascular therapeutic system capable of simultaneously promoting regeneration and preventing injury.

**Here are the key findings of our study on the effects of GLW natural extracts in promoting cardiomyocyte differentiation and cardiac repair:**

1. GLW Extracts Enhanced Cardiomyocyte Differentiation from mESCs
2. Activation of Cardiac Development Pathways Through Wnt Signaling Modulation
3. *in vivo* Cardioprotective and Functional Improvement After Myocardial Infarction
4. Confirmation of Efficacy Through Advanced Tracking and Validation Methods

## Data Availability

The original contributions presented in the study are publicly available. This data can be found here: https://www.ncbi.nlm.nih.gov/search/all/?term=PRJNA1354804.
