# Supplementary material for: Screening and characterization of natural extracts as dual-functional regulators for cardiomyocyte regeneration and cardiac repair
Source: Front Cardiovasc Med. 2025 Nov 13;12:1701482. doi: 10.3389/fcvm.2025.1701482 (PMC12657391; doi:10.3389/fcvm.2025.1701482)
Supplement: Supplementary file 2 [file Datasheet1.pdf]

Supplementary Material

1 Supplementary Figures and Tables

1.1 Supplementary Figures

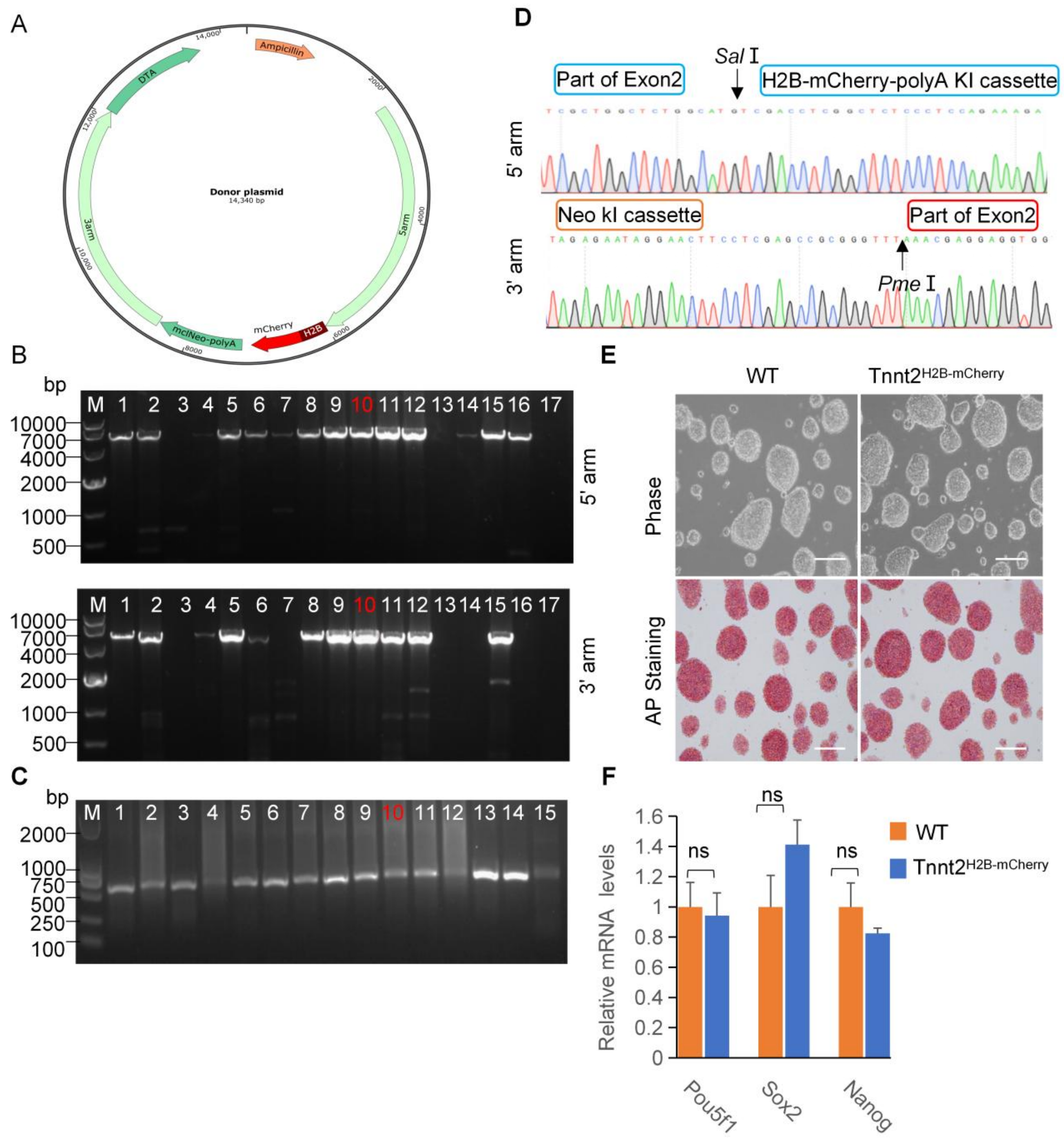

Supplementary Figure 1.

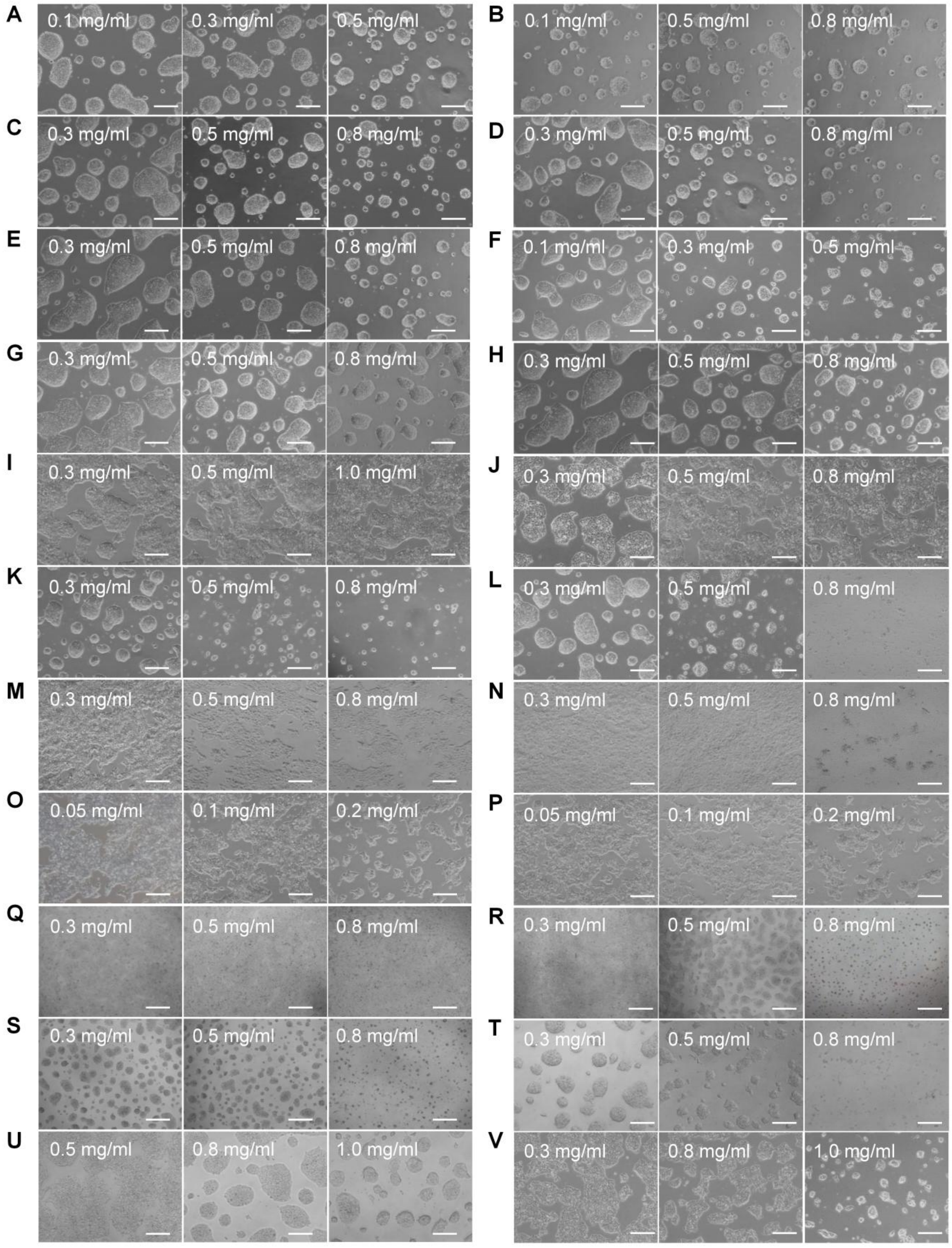

Supplementary Figure 2.

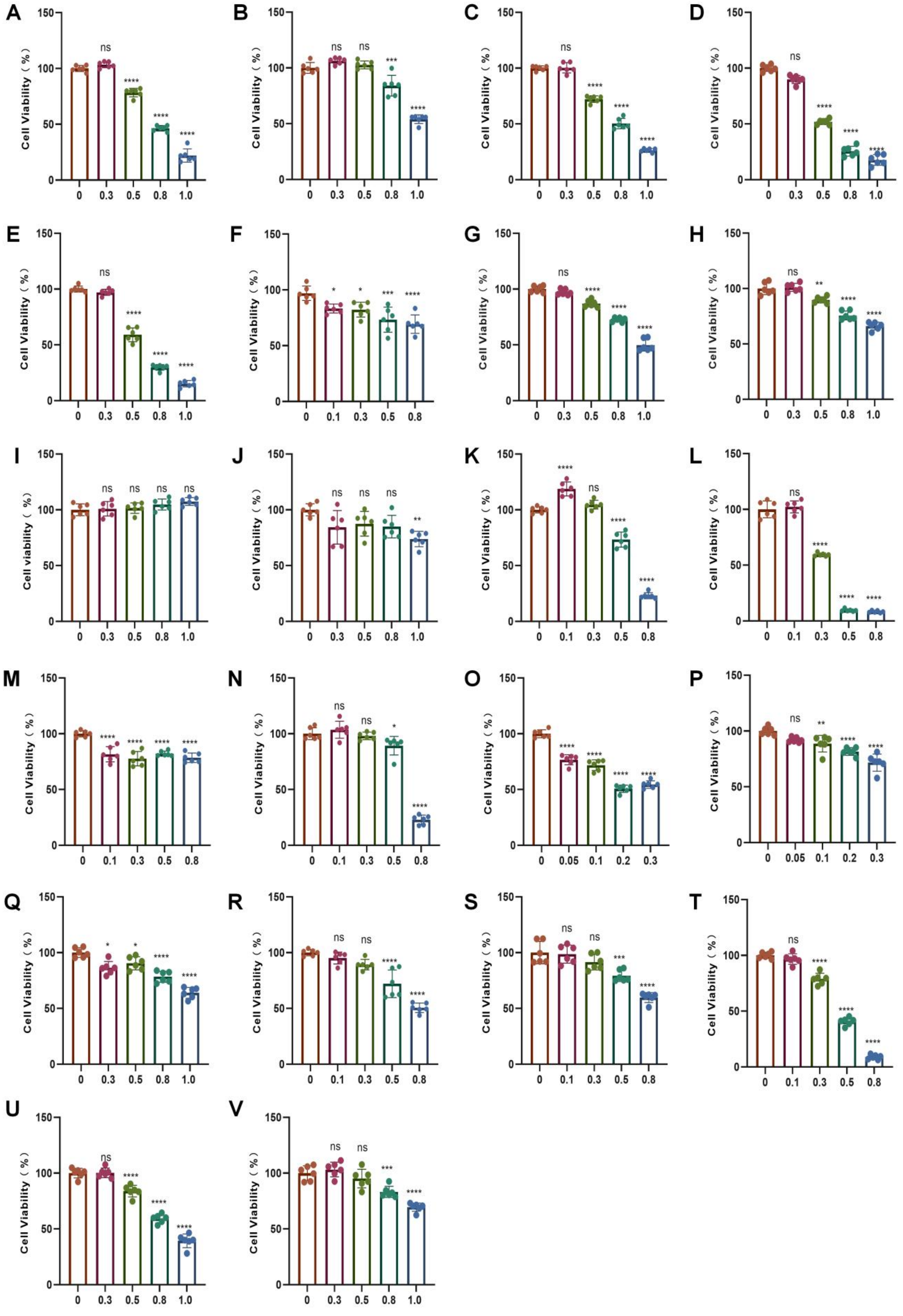

Supplementary Figure 3.

2.1 Supplementary Tables

Table S1.Information about the natural extracts used in this study

ID	Natural product	abbreviation	Lot number	concentration（mg/ml）
1	*Hypsizygus marmoreus*	HM	B1-2	0.3
2	*Lyophyllum decastes*	LD	E1-2	0.5
3	*Lyophyllum decastes* root	LDR	E2-2	0.3
4	*Tetrastigma hemsleyanum*	TH	SYQ210118-1	0.3
5	*75% ethanol-extracted Ganoderma lucidum spores*	EGLS	TQ02-220703	0.3
6	*Ganoderma lucidum Water extractum*	GLW	LZ20220301	0.1
7	*sporoderm-removed Ganoderma lucidum spores*	RGLS	20220401	0.3
8	*sporoderm-broken Ganoderma lucidum spores*	BGLS	LB-2021	0.3
9	*Dendrobium officinale*	DOP	YP240104005	0.8

	polysaccharide			
10	*Dendrobium officinale extractum*	DOE	YP240904001	0.8
11	*American ginseng radix saponins*	AGRS	YP240603011	0.1
12	*American ginseng saponins*	AGS	YP230728030	0.2
13	*Ganoderma lucidum alcohol precipitate*	GLAP	YP240603008	0.1
14	*Ganoderma lucidum alcohol precipitatio supernatant*	GLAPS	YP240603007	0.3
15	*Cordyceps flos alcohol extractum*	CFAE	YP221130003	0.05
16	*Cordyceps flos water extractum*	CFWE	YP221130002	0.05

18	*Hericium erinaceus extractum*	HEE	YP241106002	0.3
19	*Gynostemma pentaphyllum saponin extractum*	GPSE	YP240904002	0.3
20	*Ginseng radix saponins*	GRS	YP231120001	0.1
21	*Ginsenosides*	G	YP230728028	0.1
22	*Panax notoginseng radix saponin*	PNRS	YP231120004	0.3
23	*Panax notoginseng caule et folium saponin*	PNCEFS	YP231120003	0.5

Supplementary Table 1.

Table S2. Primers used in this study

Gene	Primer Sequence (5' to 3')	PS (bp)
SgRNA-*Tnnt2*	F-GCTCGGCGTCAGACATGCTCT	20
	R- AGAGCATGTCTGACGCCGAGc	
Tnnt2 F1+ R1	F-CAGTCCCTGTTCAGAGGTAAGACA	5649
	R-TAAGATACATTGATGAGTTTGG	

Tnnt2 F2+R2	F- GCTGTGCTCGACGTTGTCAC	4863
	R-GTGACAGGACATCAAGACTCACTG	
β-actin	F- TAGGCACCAGGGTGTGATGG	282
	R-ATGGCTGGGGTGTTGAAGG	
Pou5f1	F-GGCTAGAGAAGGATGTGGTTCGAG	118
	R-CCTGGGAAAGGTGTCCCTGTAG	
Nanog	F-TGAGCTATAAGCAGGTTAAGAC	136
	R- CAATGGATGCTGGGATACTC	
Sox2	F-CGGCACAGATGCAACCGAT	86
	R-CCGTTCATGTAGGTCTGCG	
Brachyury	F- CTCGGATTCACATCGTGAGAG	148
	R-AAGGCTTTAGCAAATGGGTTGTA	
Isl1	F- AAGGACAAGAAACGCAGCAT	85
	R-TTCCTGTCATCCCCTGGATA	
Tnnt2	F- GTAGAGGACACCAAACCCAAG	139
	R-GAGTCTGTAGCTCATTCAGGTC	
Myh6	F- GATGCCCAGATGGCTGACTT	275
	R- GGTCAGCATGGCCATGTCCT	
Myl7	F- CCCATCAACTTCACCGTCTTCCT	167

	R- AGAGAACTTGTCTGCCTGGGTCA	
Mef2c	F- ATCCCGATGCAGACGATTCAG	132
	R- AACAGCACACAATCTTTGCCT	
Gata4	F- CTCTATCACAAGATGAACGGCATCAAC	100
	R-TCTGGCAGTTGGCACAGGAGAG	
CTNNB1	F-ATGGAGCCGGACAGAAAAGC	143
	R-TGGGAGGTGTCAACATCTTCTT	
Axin1	F-CATTGTGTCCAGACAAACCAAGC	139
	R-GGAAGGGTAGGTATTCTCCTCCA	
Wnt3a	F-CAGGAACTACGTGGAGATCATGC	210
	R-CGTGTCACTGCGAAAGCTACT	
Wnt5a	F-ATGCAGTACATTGGAGAAGGTG	138
	R-CGTCTCTCGGCTGCCTATTT	

PS, product size; F, forward; R, reverse

Supplementary Table 2.
